# Comparative Genomic Analyses of Escherichia coli from a Meat Processing Environment in Relation to Their Biofilm Formation and Persistence

**DOI:** 10.1128/spectrum.00183-23

**Published:** 2023-05-15

**Authors:** Xianqin Yang, Frances Tran, Peipei Zhang

**Affiliations:** a Agriculture and Agri-Food Canada, Lacombe, Alberta, Canada; The Pirbright Institute

**Keywords:** biofilm, curli, cellulose, persistence, antimicrobial resistance genes

## Abstract

We investigated the phylogeny of biofilm forming (BF) and nonbiofilm forming (NBF) Escherichia coli (*n* = 114) from a beef processing environment as well as genetic elements in their BF and persistence via a comparative genomic analysis. Phylogroup B1 made up the largest proportion of both the BF (73.8%) and NBF (50.9%) groups. E. coli from all of the sources that were examined had mixed phylogroups, except for those that were recovered from equipment after cleaning, which were exclusively from phylogroup B1. Both the core genome and gene content trees showed a tree-wide spread of BF strains, with clusters, including both BF and NBF strains. Genome-wide association studies (GWAS) via Scoary or Pyseer did not find any genes or mutations that were overrepresented in the BF group. A retrospective analysis of phenotypes found a significant correlation (*P* < 0.05) between BF ability and curli production, cellulose synthesis, and/or mobility. However, the BF group also included strains that were negative for curli and cellulose and/or missing encoding genes for the two traits. All curli and cellulose encoding genes were present in most genomes, regardless of their BF status. The degree of motility was correlated with both curli and cellulose production, and 80 common genes were overrepresented in all three of the trait-positive groups. A PTS enzyme II, a subsidiary gluconate catabolism pathway, and an iron-dicitrate transport system were more abundant in the persisting E. coli group. These findings suggest gene function redundancy in E. coli for biofilm formation as well as additional substrate utilization and iron acquisition in its persistence.

**IMPORTANCE** The persistence of potentially hazardous bacteria is a major challenge for meat processing environments, which are conducive for biofilm formation. Marker genes/phenotypes are commonly used to differentiate biofilm forming E. coli strains from their nonbiofilm forming counterparts. We took a comparative genomic analysis approach to analyze E. coli strains that were from the same environment but were differentiated by their biofilm forming ability. A diversification of the genes involved in the biofilm formation of E. coli was observed. Even though there is a correlation on the population level between biofilm formation and the expression of curli and cellulose, uncertainties exist on the individual strain level. Novel substrate utilization and iron acquisition could contribute to the persistence of E. coli. These findings not only advance our understanding of the ecology of E. coli with respect to its persistence but also show that a marker gene/phenotype driven approach for the biofilm control of E. coli may not be prudent.

## INTRODUCTION

Biofilms are aggregated microbial communities that are attached to biotic or abiotic surfaces and are typically embedded in self-produced extracellular polymeric substances (EPS) (1). They are characterized by an increased tolerance to stress, which leads to the survival of microorganisms in adverse environments, including meat processing facilities, where it is common to have conditions such as extreme temperatures, desiccation (e.g., equipment surfaces during downtime), and the application of biocides for the cleaning and sanitization of processing equipment and of antimicrobials as interventions for reducing the contamination of products (2, 3). Recent work has demonstrated that biofilm formation plays a more important role in the persistence of Escherichia coli on beef processing equipment, compared to the resistance of planktonic cells to biocides (4, 5). E. coli strains from equipment surfaces are the primary sources of contamination of beef in some facilities (6, 7). Shiga toxin-producing E. coli, particularly the serotype O157:H7, can cause severe intestinal infections in humans, and these infections may further develop into hemolytic-uremic syndrome and even death (8). However, they do not cause symptoms in cattle, even though they colonize the lower portion of the colon (the recto-anal junction) of cattle (9), which makes the organism a significant food safety hazard in beef processing. E. coli O157:H7 is normally found on beef at low prevalence and at low numbers when it is present (10). However, beef processing plants may experience sporadic peaks of contamination of beef trimmings with E. coli O157:H7 that are clustered in a short time frame (11), and this phenomenon has been linked to the biofilm formation and the ensuing resistance to sanitizers of some E. coli O157:H7 strains (12).

Biofilm development is marked by a few discrete steps: initial, reversible attachment of planktonic cells to a surface, irreversible attachment, microcolony growth, maturation, and dissolution, which releases bacterial cells back into the planktonic state, and the entire process is a highly coordinated network of interactions of the bacterial cells and environmental cues (13). For E. coli, numerous studies have focused on functions of genes that are involved in biofilm formation, mostly based on the mutation of a single gene or a gene operon and the characterization of the resulting phenotype (14). For instance, it has been demonstrated that the fimbrial adhesion curli, which is encoded by the *csg* operon, and cellulose production, the synthesis of which is controlled by two divergently transcribed operons, namely, *bcsABCD* and *bcsEFG*, are essential for the biofilm formation by E. coli (15, 16). These two features have also been widely used to evaluate the biofilm forming capability of E. coli and other members of *Enterobacteriaceae*, based on differential colony staining by the azo-dye Congo red, which semispecifically binds to amyloid structures, including curli and cellulose (17, 18). A positive correlation between the total numbers of these two positive traits in dual-species cultures and their degree of biofilm formation has been reported (19). Even so, the assessment of the applicability of these features on a population level for E. coli is lacking.

The primary habitat for E. coli is the intestines of warm-blooded animals; however, the organism is versatile and can adapt to various secondary environments (20). This versatility is largely attributable to its ability to acquire genes through horizontal gene transfer (HGT) (21). Antimicrobial resistance (AMR) genes in E. coli are often located on mobile genetic elements and are consequently, transmitted through HGT events (22). Biofilms not only enhance bacterial tolerance to antibiotics through their physiochemical properties but also may contribute to AMR by providing a much more conducive environment for HGT, compared to that of their free living planktonic counterparts (23). In addition, E. coli strains have been historically grouped into seven major phylogroups: A, B1, B2, C, D, E, and F (24). Phylogenetic group specific carriage of certain traits and survival in different ecological niches have been reported for E. coli. For instance, the phylogroup B1 E. coli has a much higher survival rate and prevalence in fresh water, compared to other phylogroups (21, 25). The distribution of a genomic island, namely, the transmissible locus of stress tolerance (tLST), is primarily concentrated in phylogroup A E. coli (26). Information on the relationship between the phylogenetic background and the biofilm forming ability of E. coli seems largely lacking.

Our recent work has investigated the biofilm forming ability of E. coli (*n* = 700) recovered from beef processing environments and has found that the proportion of biofilm forming strains in each population varied significantly among different processing stages (27). The present study focuses on genomic analyses of selected E. coli strains with different biofilm forming abilities and persistence, aiming to discern genetic differences between these E. coli populations as well as the association of biofilm formation and commonly used phenotypic indicators (curli, cellulose, and motility).

## RESULTS

**FIG 1:**
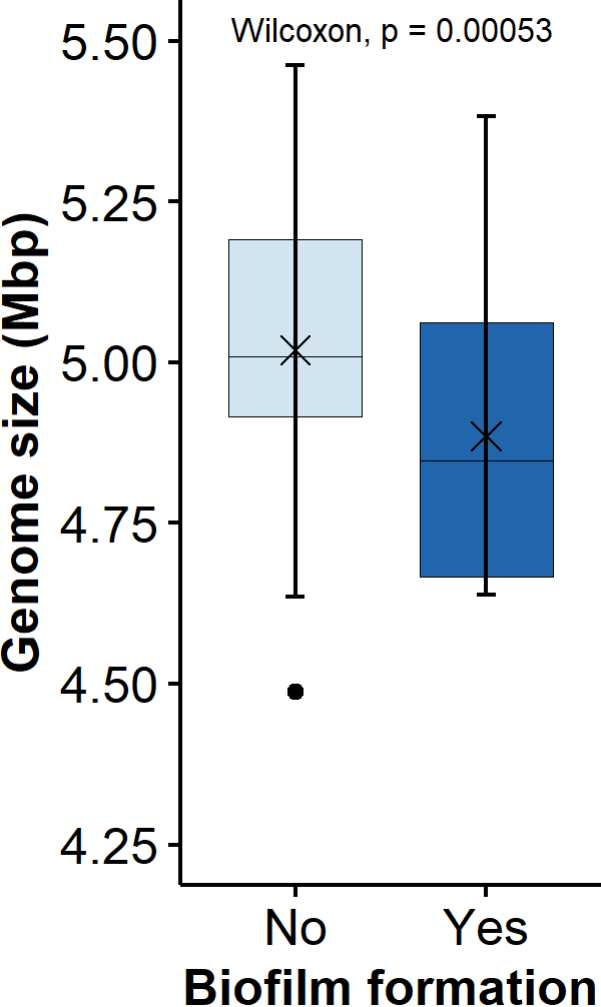
Boxplot comparing the genome size of biofilm forming (*n* = 61; Yes) and nonforming (*n* = 53; No) E. coli. The mean and median are represented with an “x” and a horizontal line, respectively. A paired Wilcoxon test was performed to compare the median values.

**FIG 2:**
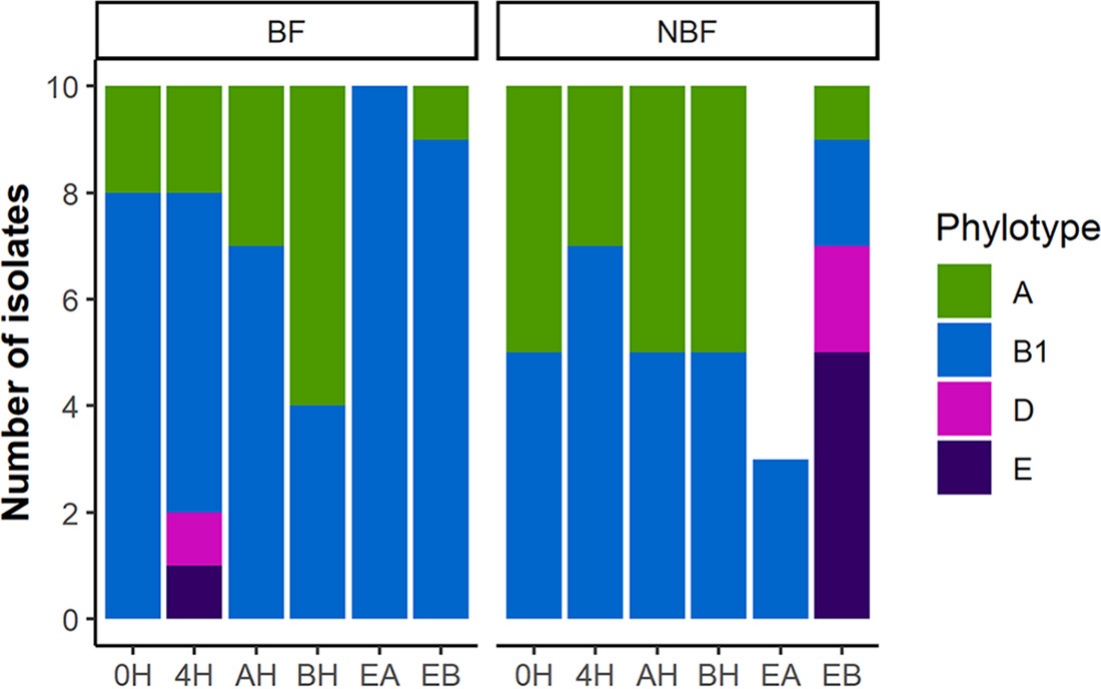
Population structure of E. coli from various sources, as determined by the ClermonTyping method. Biofilm forming (BF) and nonbiofilm forming (NBF) E. coli were isolated from beef carcasses before chilling (0H), after chilling (4H), before hide-on wash (BH), after hide-on wash (AH), and from fabrication equipment before cleaning (EB) and after cleaning (EA).

The 114 E. coli isolates that are included in this study are comprised of isolates collected from various stages of processing at two federally inspected beef packing plants (A and B). They were recovered from six different sources: beef carcasses before (BH) and after a hide-on wash (AH) at Plant A, beef carcasses before (0H) and after chilling (4H), and fabrication equipment before (EB) and after the daily routine equipment sanitation/before work started the next day (EA) at plant B. The isolates from each source had an equal number (*n* = 10) of BF and NBF strains, except for the EA group, for which only three NBF strains were available. An additional extremely strong biofilm forming E. coli strain (136) from a sanitized equipment surface was also included. The assembled draft genome size and G+C content were between 4.49 and 5.46 Mb and between 50.3 and 50.9%, respectively (Table S1). The mean genome size for the BF and NBF E. coli populations was 4.89 and 5.02 Mb, respectively, and the median values for the two groups differed (*P* < 0.001) significantly (Fig. 1). The 114 isolates belonged to four phylogroups: A (28.9%), B1 (63.2%), D (2.6%), and E (5.3%). Phylogroup B1 made up 73.8% and 50.9% of the BF and NBF groups, and this was followed by phylogroup A at 23.0% and 35.9%, respectively (Table S1; Fig. 2). Interestingly, all EA isolates, regardless of their biofilm forming capabilities, were of the B1 phylogroup, unlike the isolates in all other groups, which belonged to more than one phylogroup.

Among the 114 isolates, 44 multilocus sequence typing (MLST) types were identified, based on the 7-gene Achtman’s scheme (Table S1), and 7 of these were found to be in both the BF and NBF groups (MLST 10, 46, 58, 70, 101, 345, and 906). BF strains (*n* = 61) belonged to 21 different MLST types (strain/type ratio of 2.9), whereas more variability was observed for the NBF strains: 30 MLST types among 53 strains (strain/type ratio of 1.8). Overall, of the 44 MLST types, 8 included 5 or more isolates.

**FIG 3:**
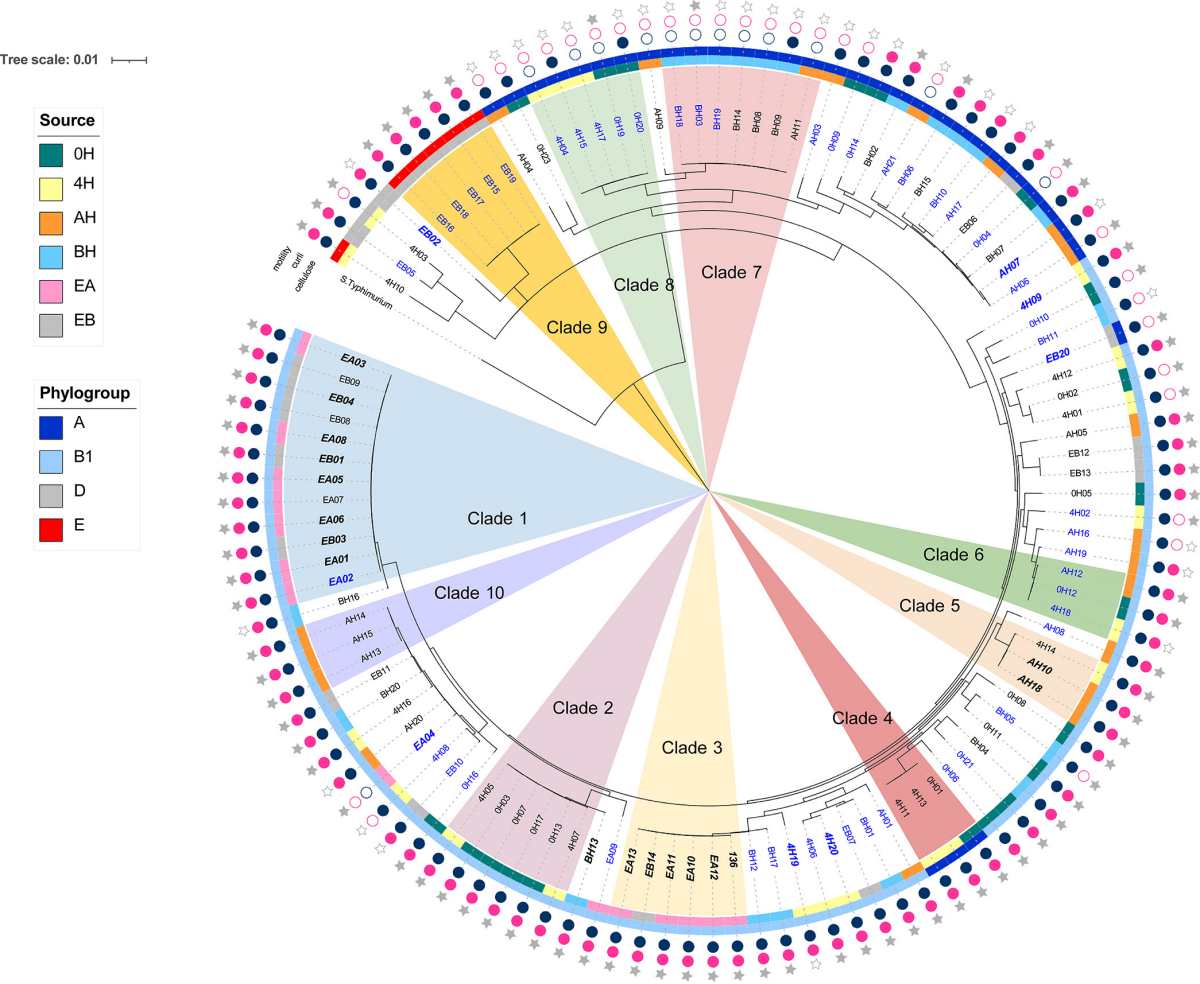
Core genome phylogenetic tree of E. coli (*n* = 114). The tree, which was constructed based on the concatenated alignment of nucleotide sequences of core genes (*n* = 3,243), was rooted using the genome of Salmonella enterica subsp. *enterica* serovar Typhimurium str. LT2 (Assembly accession no. in NCBI: GCF_000006945.2) as an outgroup. The sources of isolation and phylogroups are color-coded and shown in the first and second rings, respectively. Isolates that are labeled in black and blue are strong biofilm formers and nonbiofilm formers, respectively. Isolates that are bold, italicized, and in a slightly larger font (*n* = 25) were persisting strains. The tree was displayed and annotated using iTOL v5 (https://itol.embl.de/).

A pangenome analysis of the 114 E. coli genomes generated 3,243 core genes. The core genome tree showed the intermingling of BF and NBF strains (Fig. 3). Clades were regarded as the first nodal point of the most recent ancestral population with more than two isolates, and there were 10 such clades identified (Fig. 3). Interestingly, clades consisting exclusively of BF (clades 2, 3, 4, 5, and 10), exclusively of NBF (clades 6, 8, and 9), or of both BF and NBF (clades 1 and 7) were found. Of the 34 total equipment isolates, the majority (*n* = 23) were found in clades 1, 3, and 9, and these clades exclusively included equipment isolates. The within clade genetic distance from the core genome MLST for each of the three equipment clades was ≤1, ≤7, and ≤3 alleles, respectively (Table S2). A larger genetic distance was observed for clade 7 at ≤82, clade 4 at ≤213, and clade 5 at ≤368 alleles (Table S2). In general, smaller genetic distances were observed for isolates from the same source where there was clustering.

**FIG 4:**
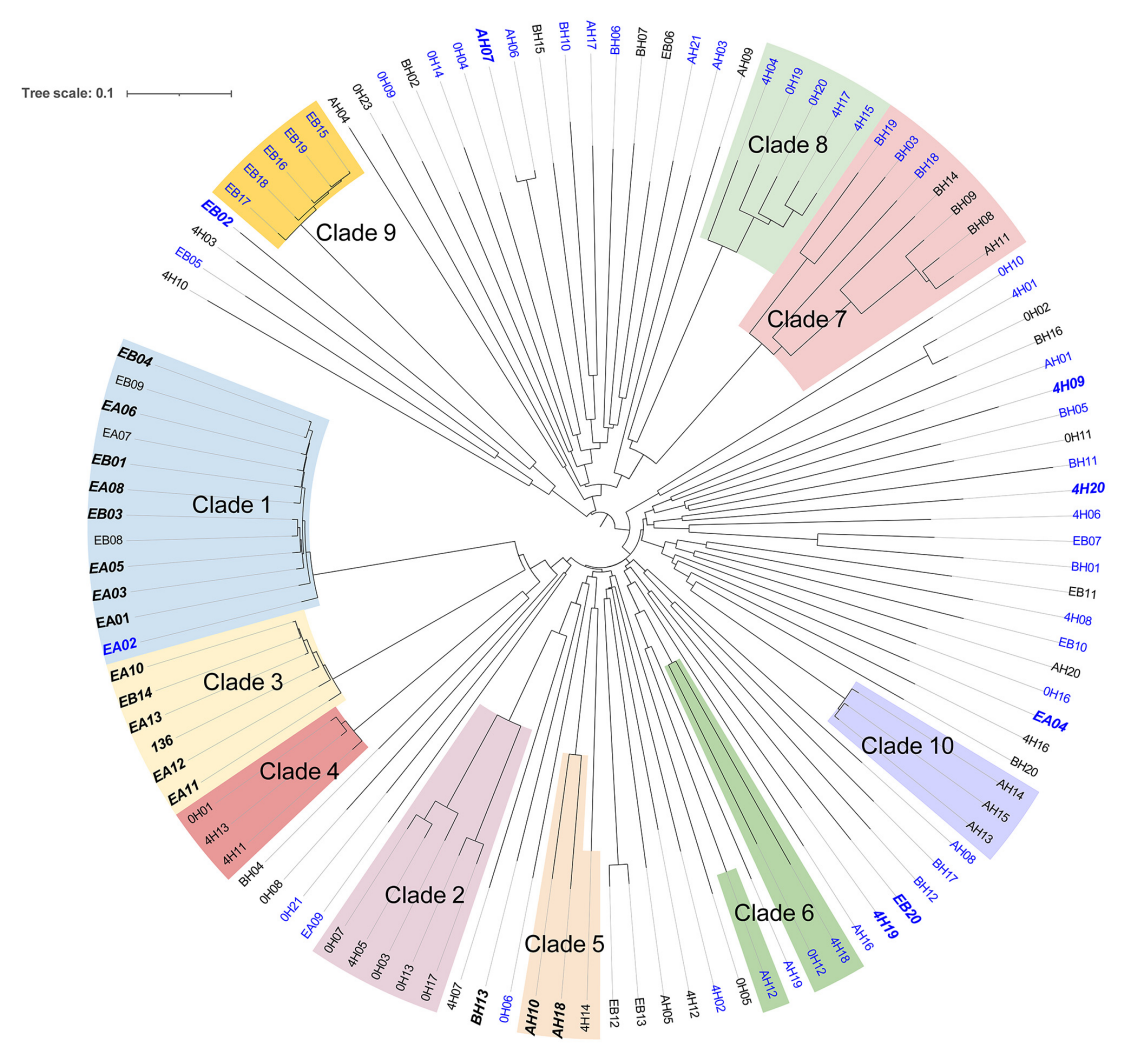
A neighbor-joining tree, built upon the accessory genome of E. coli (*n* = 114). The tree was constructed, based on the Jaccard distance, using the Phangorn package in R. Isolates that are labeled black and blue are strong biofilm formers and nonbiofilm formers, respectively. Isolates that are bold, italicized, and in a slightly larger font (*n* = 25) are persisting strains. The clades in Fig. 3 are relabeled in the same colors. The tree was midpoint displayed and annotated using iTOL (https://itol.embl.de/).

As with the core genome tree, the accessory genome tree had BF and NBF strains intermingled throughout the tree (Fig. 4). The composition and close clustering of equipment isolates (clades 1, 3 and 9) were maintained, even though slightly better refined branches were observed with the accessory genome tree. More than 50% of the persisting strains were in clades 1 and 3, and the remaining strains were spread out through both the core and accessory genome trees. All persisting strains were phylogroup B1, except for three (AH07, EB20, and EB02) which were phylogroup A or D. No clear separation of the gene content of the isolates was observed when grouping by biofilm formation (Fig. S1A), isolation source (Fig. S1B), or persistence (Fig. S1D) on the principal components analysis (PCA) plots, with grouping by phylogroup being an exception (Fig. S1C). The EA and persisting strains had a much smaller dispersion, compared to other groups (Fig. S1B and D), which corroborates with their phylogeny analysis by core and accessory genes.

Four genes were significantly associated with the BF group, as determined by Scoary: *yehD*, *yehC* and group_1227 (an arbitrary gene ID assigned in pangenome by Roary) encoding a putative fimbrial protein or a related function, namely, *yeeJ*, encoding an inverse autotransporter adhesin. Significantly overrepresented kmers were mapped to six genes (*mpl*, *truB*, *idnD*, *idnO*, *idnR*, and *idnT*). However, none of these genes met the criteria of present in >60% of the BF group but absent in > 60% of the NBF group. There were 58 genes overrepresented in the persisting E. coli group, 34 of which were related to mobile element functions, such as genes encoding transposases, prophages, and phage genes (Table S3). In addition, two complete operons encoding a putative PTS enzyme II (*sgcABC*) and a subsidiary gluconate catabolism pathway (*idnROKDT*), respectively, and genes that were part of an iron-dicitrate transport system (*fecB* and *fecD*) were overrepresented in the persisting group. Interestingly, the *idn* operon was present in 27 of the 34 isolates from equipment, compared to the 15/80 carcass isolates.

**FIG 5:**
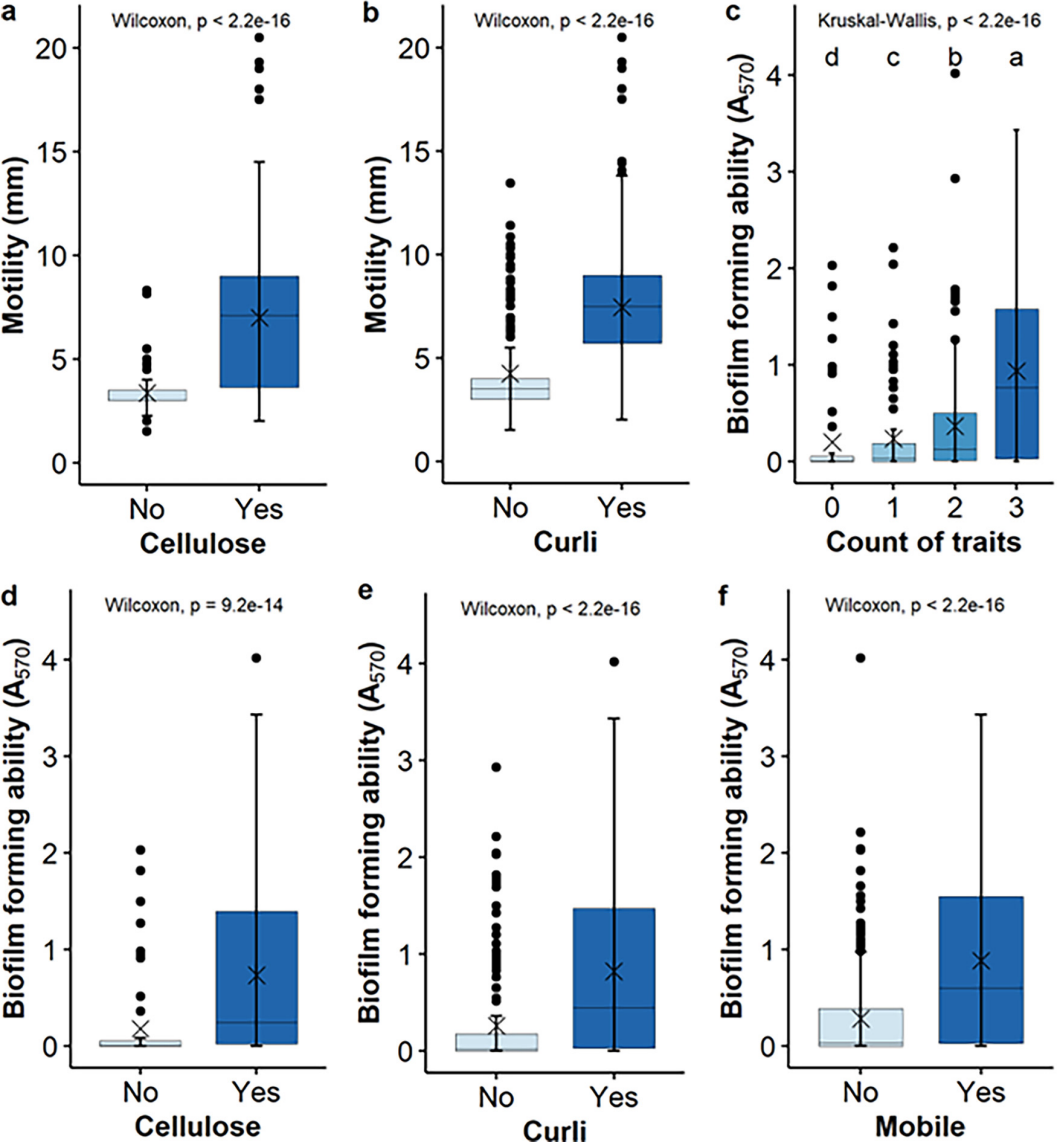
Correlation between the expression of curli, cellulose, and motility (A and B) as well as between the biofilm formation and these determinants individually (D–F) and cumulatively (panel C) in E. coli (*n* = 700).

There were 86, 92, and 177 genes overrepresented in the E. coli group that either were motile or expressed curli or cellulose phenotypes. Interestingly, 80 genes were common among all three trait-positive groups (Table S4–S6), which is consistent with the phenotypic characterization (i.e., 71 of the 105 isolates that were positive for at least 1 trait were positive for all 3). The (predicted) functions of these common genes included the CRISPR-associated proteins (CAS), adhesins, novel sugar/carbohydrate utilization, secretion system, transcription regulators, transport system, and stress response. Of the six additional overrepresented genes in the motile group, four were involved in survival/intercellular competition (*RhsB*, *DedA*, *LdrA*, *MdtO*) and the remaining two were involved in carbohydrate degradation (Table S4). Among the 12 additional overrepresented genes in the curli group are those involved in biofilm formation, such as *fimA*, *pgaA*, *fliD*, and *csrD*, as well as a gene encoding a two-component LuxR transcriptional regulator (Table S5). Different from the small number of additional overrepresented genes in the curli and motility positive groups, the cellulose positive group had 97 such genes, including those encoding adhesins (*n* = 10), the entire operon for colanic acid production (*n* = 20), the DNA-binding transcriptional activator MlrA of the master biofilm regulator *csgD*, and a cyclic di-GMP phosphodiesterase (*pedR*). The products of these genes (*n* = 31) have been predicted/reported to be involved in biofilm formation. The additional overrepresented genes in the cellulose positive group also included stress resistance/survival (e.g., *marB* and *mdtO*) and novel substrate utilization (e.g., a complete *hpa* operon). Interestingly, the degree of motility and the expression of curli or cellulose was positively correlated (Fig. 5A and B). The biofilm forming capability of the isolates was positively correlated with the expression of curli, cellulose, or motility, and it increased with an increasing of number of determinants (Fig. 5C–F). Even so, it is noteworthy that biofilm formers were also found in strains that were negative for any one of the three determinants or all three of the determinants.

**FIG 6:**
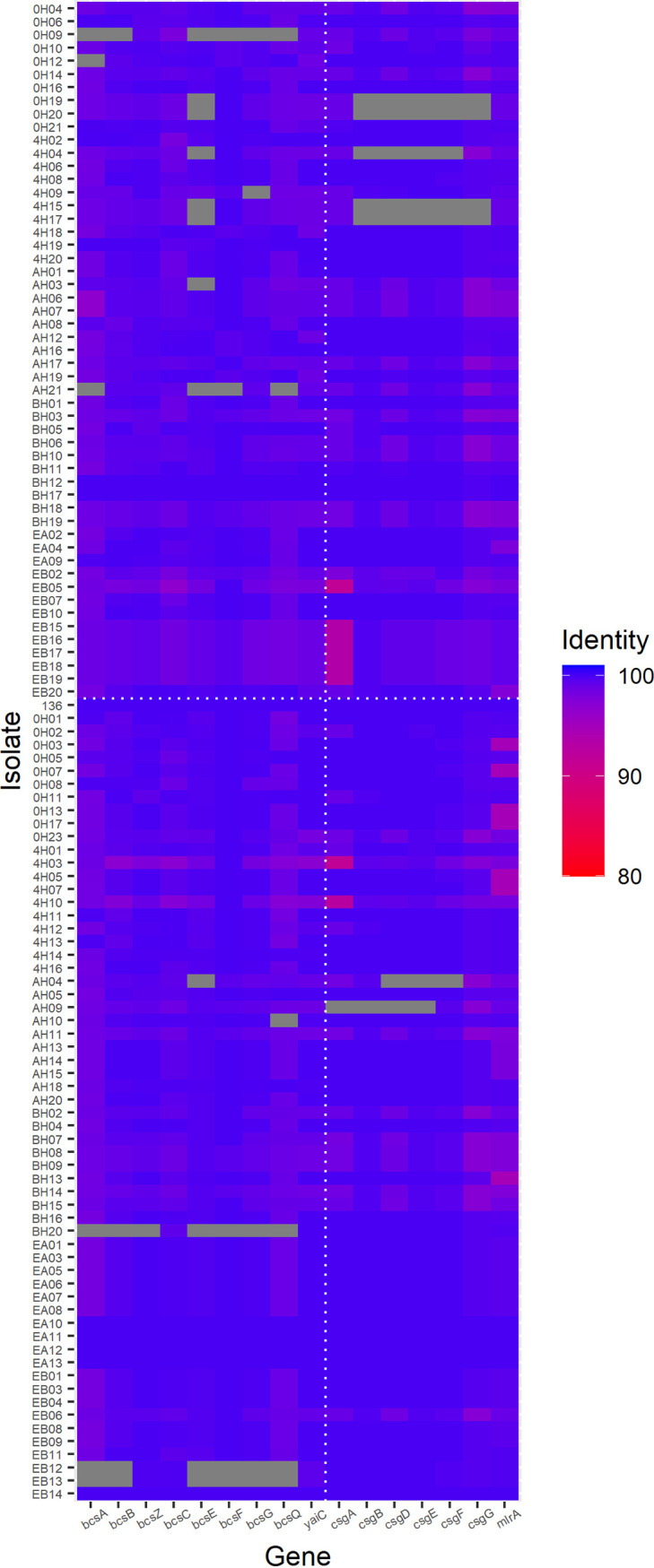
The presence and extent of the similarity of genes involved in cellulose and curli production in the E. coli genomes (*n* = 114). The vertical dotted line separates the genes that are involved in cellulose (left) and curli (right) biosynthesis. A horizontal dotted line separates the nonbiofilm formers (top) from the biofilm formers (bottom). The identities between relevant genes in each isolate and in strain 136 are shown in color from red to blue. The grids filled with gray indicate that the isolate has zero copies or more than one copy of the corresponding gene; hence, an identity level is not shown.

All genes involved in the biosynthesis of cellulose and curli were present in the majority of the 114 genomes, with only 13% (*n* = 15) and 6% (*n* = 7) missing one or more genes of each operon but never the entire respective operons (Fig. 6). The BF group included genomes that were missing genes in the biosynthesis of curli (*n* = 2), cellulose (*n* = 5), or both cellulose and curli (*n* = 1). On the other hand, 43 genomes (out of 53) from the NBF group did not lack any genes in either curli or cellulose generation. Some isolates that exhibited a positive phenotype for cellulose production lacked one or more genes involved in its biosynthesis, whereas all isolates positive for curli formation had all of the genes required for curli production (Fig. S2).

In total, 8 and 11 distinct genes related to antimicrobial resistance (AMR) were found in the BF and NBF groups, and they were distributed in 26.2% (16/61) and 39.6% (21/53) of their respective groups. These genes were related to aminoglycoside, β-lactam, phenicol, fosfomycin, sulfonamide, and tetracycline resistance (Fig. S3). Of the isolates from carcasses before intervention (BH and 0H), after intervention (AH and 4H), and from equipment, 42.5% (17/40), 40.0% (16/40), and 11.8% (4/34) carried one or more antibiotic resistance genes.

## DISCUSSION

The meat processing environment includes multiple ecological niches, from hides of animals representing the rearing and transportation conditions to fabrication equipment surfaces, which are exposed to low temperatures and cleaning agents (2, 3). These different environmental conditions may shape E. coli populations, in relation to their biofilm formation and persistence. In the present work, we used a population approach to examine the relationship of phylogenetic background and biofilm formation in E. coli and to explore the genetic determinants of their biofilm formation and persistence.

The MLST analysis, cgMLST analysis, and core genome phylogeny showed a similar trend in the clustering and intermingling of BF and NBF strains in the same subtype or lineage. These findings suggest that the BF and NBF strains may not always differ in their core genes or genes used for subtyping. As such, assessing the biofilm forming ability of E. coli strains may not be as distinctive through subtyping. The lack of clear separation of gene content on the PCA plot in the present study suggests that different subsets of genes may be involved in the biofilm formation of different E. coli populations. Thus, it is not surprising that GWAS, using the criteria of being present in 60% of the BF group and absent in 60% of the NBF group, failed to identify any genes that were overrepresented in the BF group. However, it is noteworthy that the sample size of this study is relatively small for GWAS. The outcome of GWAS is affected by the sample size, and increasing the sample size will improve the power of GWAS to recover meaningful associations, especially when traits are affected by multiple genes with small effects. These findings are in concordance with a study of Fang and colleagues (19), which compared six STEC strains for their biofilm forming ability, curli and cellulose production, and distribution of genes potentially involved in biofilm formation and found a redundancy of genes involved in all of the different stages of biofilm formation.

The present study also showed that the majority of genomes harbored genes involved in cellulose and curli production, regardless of their biofilm formation status. More interestingly, strains missing one or more genes in the biosynthesis of both extracellular structures were able to produce strong biofilms. This result is also corroborated by findings of strains that did not produce curli or cellulose but did form strong biofilms. In addition to curli and cellulose, the azo-dye Congo red may also bind to the exopolysaccharide poly-β-1,6-*N*-acetylglucosamine (28) and the autotransporter adhesion YeeJ (29), which could explain the cellulose positive strains with missing cellulose encoding genes. An engineered E. coli strain with 17.6% of the parental genome removed, including the genes involved in the synthesis of various cell structures, such as type I fimbriae, curli, exopolysaccharide polymers, and the autoinducer-2 signal molecule, is able to develop mature biofilms (30). These findings point toward the diversification of genes required for functions in E. coli biofilm formation. Thus, gene expression studies based on the mutation or deletion of single genes or gene clusters provided significant value toward understanding their roles in biofilm formation in individual strain. Further systems studies on how different sets of genes that would result in similar physiological functions for biofilm formation are warranted.

Bovine-associated commensal E. coli consist mainly of strains belonging to phylogroups A, B1, and small fractions of phylogroups C, D, and E (31). The population structure of E. coli from carcasses in the present study largely reflected that of bovine commensals. However, the E. coli population from equipment after cleaning was exclusively phylogroup B1, suggesting a better survival of this phylogroup in the fabrication facilities of meat processing plants. The ambient temperature in the fabrication facility from which these isolates were collected was maintained at 6 to 7°C during production (3). Compared to various phylogroups of E. coli from animal hosts, B1 strains have longer persistence in fresh water, and those persisting strains have lower minimum growth temperatures (21, 25). Together, these findings suggest that phylogroup B1 may survive better than other phylogroups in environments where the temperature is low and nutrients are not abundant. The better survival of the B1 strains in secondary environments has been attributed to their gene repertoire of alternative carbon source metabolism (21). The present work found that genes involved in mobile element functions, complete operons encoding a PTS enzyme II (*sgcABC*) and a subsidiary gluconate catabolism pathway (*idnROKDT*), as well as genes that were part of an iron-dicitrate transport system (*fecB* and *fecD*) were overrepresented in persisting strains that were primarily phylogroup B1.

The BF group had a higher proportion of phylogroup B1 than did the NBF group (73.8% versus 50.9%) as well as a significantly smaller genome size. There is a paucity of information in the literature on the relationship between the phylogroup and the biofilm forming ability of E. coli. Smaller E. coli genomes have a higher turnover of genetic information, which is indicated by their higher rates of gene repertoire diversification, and fewer but more diverse mobile genetic elements, compared to larger genomes (21). E. coli strains from primary hosts that lost their cultivability within 4 days of incubation in estuarine water had more abundant virulence factors and antibiotic resistance genes than did those that persisted in water (25). The findings of the present study that antibiotic genes were more prevalent in strains from carcasses (approximately 40%) than those from equipment (11.8%) are consistent with the results of previous reports. However, the lower prevalence of AMR genes in the BF group (26.2%) than in the NBF group (41.5%) was contradictory, with reports stating high genetic material exchange rates in biofilms, compared to planktonic cells (23). This contradiction may reflect the differences between laboratory conditions and/or the low harborage of AMR genes in naturalized E. coli populations in meat plants.

Taken together, the findings of this study indicate that there is a diversification of genes involved in the biofilm formation of E. coli, as reflected by the topology of core and accessory genome trees, the gene content analysis via a PCA plot, and the GWAS analysis. Additional genes involved in novel substrate utilization and iron acquisition could contribute to the persistence of E. coli in meat processing environments, in addition to the previously reported biofilm forming ability. Even though there is a correlation between biofilm formation and the expression of curli and cellulose, as determined by the Congo red indicator (CRI) method, caution should be exercised when using these phenotypic features and their encoding genes for the assessment of biofilm forming capability on individual strain level. Instead, a phenotypic assessment of biofilm formation under relevant conditions should be included.

## MATERIALS AND METHODS

### Bacterial strain selection and culture conditions.

A total of 114 E. coli isolates were included in this study, and they were comprised of isolates that were collected from various stages of processing at two federally inspected beef packing plants (Plant A and Plant B) in Alberta, Canada. These E. coli isolates were recovered from six different sources: from beef carcasses before (BH) and after a hide-on wash (AH) with a NaOH solution at 55°C and along the dressing process at Plant A, which is a large beef processing plant where multiple antimicrobial interventions were implemented during carcass dressing, when the samples from which E. coli were recovered had been collected (32) as well as from beef carcasses before (0H) and after chilling (4H) and from fabrication equipment before (EB) and after the daily routine equipment sanitation/before work started the next day (EA) at Plant B, which is a small beef packing plant where no antimicrobial interventions other than the trimming and cold water washing of carcasses were employed in the carcass dressing process, and the carcasses were chilled by dry-chilling with cold air at 1°C when the samples from which E. coli were recovered had been collected (4, 6, 33). A previous study from our laboratory evaluated the biofilm forming capability of E. coli from these 6 processing stages with 100 strains for each stage via the crystal violet (CV) staining method, with the absorbance measured at 570 nm (A_570_) (27). The degree of biofilm formation of an isolate was defined based on the ratio of its absorbance of the CV solution to that of the cutoff A_570_ (A_570c_), which is defined as A_570c_ = 3 · Stdev_control_ + A_570control_, where Stdev_control_ is the standard deviation of the negative control, and A_570control_ is the absorbance value of the negative control. The nonbiofilm former (NBF) and extremely strong biofilm former (BF) groups were those with A_570_ ≤ A_570c_ and >16 · A_570c_, respectively (27). All E. coli groups included in the present study had an equal number (*n* = 10) of BF and NBF strains, except for the EA group, in which only 3 NBF were found in the 100 E. coli isolates that were screened. All three were included in the present study. In addition, an extremely strong biofilm forming E. coli strain (136) that was recovered from equipment surfaces before cleaning at plant B (6) was also included in the selection (16). These isolates were all genotyped via a multiple-locus variable number tandem repeat analysis (MLVA), and 25 of the 114 isolates were regarded as persisting, as they had been recovered multiple times over an extended period of time (4, 6, 16, 33).

Of the 114 E. coli isolates, 11 were whole-genome sequenced in a previous study (34). For the remaining 103 isolates, a −80°C stock culture of each was streaked onto MacConkey agar (Oxoid, Nepean, Ontario, Canada) that was subsequently incubated at 35°C for 24 ± 2 h. A single colony was then subcultured in 10 mL Luria-Bertani broth (LB; BD, Fisher Scientific, Ontario, Canada) for 18 ± 2 h at 35°C, with shaking at 80 rpm.

### Whole-genome sequencing.

The LB overnight cultures were subjected to DNA extraction using a Qiagen DNeasy Blood and Tissue Kit (Qiagen, Toronto, Ontario, Canada). The DNA samples of all isolates, except for strain 136, were subjected to shotgun library preparation and subsequent sequencing (PE150) using an Illumina HiSeqX platform by Genome Quebec (Montreal, Quebec, Canada). E. coli strain 136 was included in a different batch of sequencing, which used an Illumina HiSeq4000 platform (PE100).

The quality control of the raw sequencing reads was performed using FastQC v0.11.8 and MultiQC v1.8 (35, 36). Trimmomatic v0.39 was used to remove the adapter sequences as well as the sequences with average quality scores of <30 (37). Each genome was assembled using SPAdes v3.13.1 (38). The quality of assembled genomes was assessed using Quast v5.0.2 (39). Contigs with a length of <500 bp or a k-mer coverage of <10 were removed using a Python script (40). The remaining contigs were ordered using Mauve v2015-02-13 (41) by referencing the complete genome of E. coli str. K-12 substr. MG1655 (42). The ordered genomes were annotated using Prokka v1.14.6 (https://github.com/tseemann/prokka).

### *In-silico* subtyping.

The identity at the species level and the serotype of each isolate were determined using ECTyper v0.9.0 (https://github.com/phac-nml/ecoli_serotyping) with the default settings. Multilocus sequence typing (MLST) with the Achtman’s seven-gene scheme was performed using the command line tool mlst v2.19.0 (https://github.com/tseemann/mlst). The genomes were also subtyped for their phylogroup using ClermonTyper v20.03 (43). The nucleotide sequence of each genome was scanned for AMR genes using Abricate v1.0.1 (https://github.com/tseemann/abricate) as the searching tool and the ResFinder database (v2022-03-17; http://cge.cbs.dtu.dk/services/ResFinder/) as the reference.

### Pangenome and phylogenetic analysis.

The pangenome of the 114 isolates was analyzed using Roary v3.13.0, with the identity threshold for clustering proteins set as 90% (44). The core genes, which are those present in >95% of the genomes, were concatenated and subjected to maximum likelihood tree construction using RAxML v8.2.12 (45), using the general time reversible gamma nucleotide model (GTRGAMMA) and a bootstrap analysis for 100 iterations. Genes that were present in all genomes were removed from the Roary pangenome output, and the remaining genes were used to calculate the Jaccard distance via the dist() function in R, based on the presence and absence of genes. A neighbor-joining tree was constructed, based on the Jaccard distance, using the Phangorn package in R. Both core-genome and accessory genome trees were visualized and annotated in iTOL v5 (46). A core genome MLST (cgMLST) analysis was performed using ChewBBACA v2.5.6 with the default settings and the E. coli schema (2,513 genes, http://enterobase.warwick.ac.uk/schemes/). Sequences that did not meet the following criteria were removed: both valid start and stop codons were present in the coding sequence; the sequence length must be a multiple of three; and the sequence did not contain ambiguous characters (i.e., contained ATGC only). The pairwise allelic distance between E. coli isolates was calculated using cgmlst-dists v0.4.0 (https://github.com/tseemann/cgmlst-dists). A principal components analysis (PCA) of the gene contents was carried out (26). Briefly, the Roary pangenome output was used as the gene content input for the PCA analysis, using the prcomp function and the package factoextra in R, with separation by the biofilm forming ability, source of isolation, phylogroup, and persistence.

### Genome-wide association analysis.

To determine the genes that were involved in biofilm formation, in cellulose, curli, and motility phenotypes, and in persistence, a genome-wide association analysis was carried out using a previously performed phenotypic data set (27) and Scoary v1.6.16 (47). Genes with a false discovery rate of < 0.05, as indicated by Benjamini_H_p, which is a *P* value that has been adjusted via the Benjamini-Hochberg correction for multiple comparisons, and an odds ratio above 1 were selected, omitting hypothetical proteins. Genes that were present in the majority of trait-positive isolates (sensitivity of >60%) but absent in the majority of trait-negative isolates (specificity of >60%) were regarded as overrepresented in the trait-positive group.

A k-mer-based, alignment-free genome-wide association method was applied to determine whether the changes within genes were associated with biofilm formation using Pyseer v1.39 (48). Briefly, the fsm-lite command of Pyseer was used to extract k-mers with lengths between 9 and 100 bases. The k-mers with allele frequencies of ≥1% or ≤99% were tested for their association with biofilm formers by using the pyseer command of Pyseer. To correct the effect on the *P* values of the population structure of the isolates that were included in the test, a core single nucleotide polymorphism (SNP) analysis was performed using Snippy v4.6.0 (https://github.com/tseemann/snippy). A similarity kinship matrix was constructed using the similarity_pyseer command of Pyseer and used as the input for the pyseer command. The environment source of the isolates was used as a covariate in the test. The count_patterns.py of Pyseer was used to determine the significance threshold of the *P* values, which was 1.20E−08 in this study.

### Retrospective analysis of biofilm formation and the expression of curli, cellulose, and motility.

The motility as well as the curli and cellulose production of the 700 E. coli strains from which the strains in the present study were selected were assessed (27). For curli and cellulose, overnight LB cultures were streaked on Congo red indicator agar and incubated at 15°C for 4 days. The phenotypes of the colonies on CRI plates were regarded as positive for both cellulose and curli, for curli, for cellulose, or for neither if they were red, brown, pink, or white, respectively. For motility, 1 μL of a 100-fold dilution of overnight LB cultures was point-inoculated into the center of tryptone yeast extract agar and was incubated at 15°C for 48 h. Isolates that produced a motility halo of ≥4 mm in diameter were classified as motile.

The A_570_ values of the groups of isolates that were associated with the expression or lack of curli, cellulose, and motility, either individually or cumulatively, were compared using the Wilcoxon rank-sum test for comparisons between two groups (A_570_ versus curli or cellulose production) or the Kruskal-Wallis test for comparisons among multiple groups (A_570_ versus counts of traits). In addition, the correlation between the degree of motility and the production of curli or cellulose was assessed using the Wilcoxon rank-sum test. A significance level of *P* < 0.05 was used for all of the statistical analyses.

### Data availability.

The 103 genomes that were sequenced in the present study were deposited under the BioProject PRJNA819951 (Table S1).
