# Supplementary material for: Comparative Genomic Analyses of Escherichia coli from a Meat Processing Environment in Relation to Their Biofilm Formation and Persistence
Source: Microbiol Spectr. 2023 May 15;11(3):e00183-23. doi: 10.1128/spectrum.00183-23 (PMC10269509; doi:10.1128/spectrum.00183-23)
Supplement: Supplemental file 2 — Fig. S1-S3. Download spectrum.00183-23-s0001.docx, DOCX file, 0.9 MB [file spectrum.00183-23-s0001.docx]

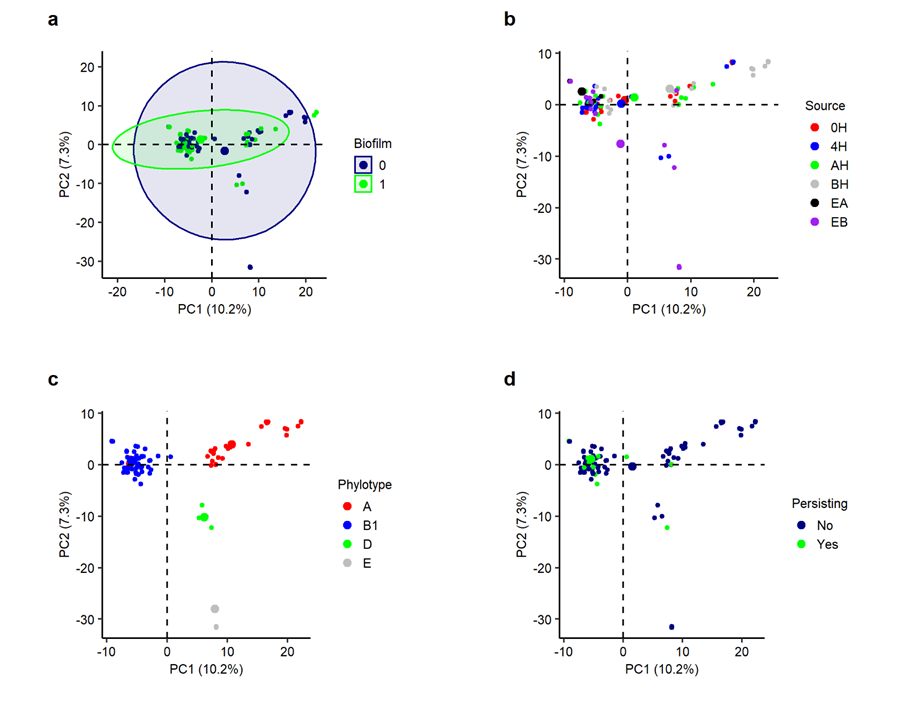


**Figure S1**.­ Principal component analysis (PCA) of gene content of the 114 *E. coli* genomes grouped by biofilm formation (a), isolation source (b), phylogroups (c), and persistence (d).


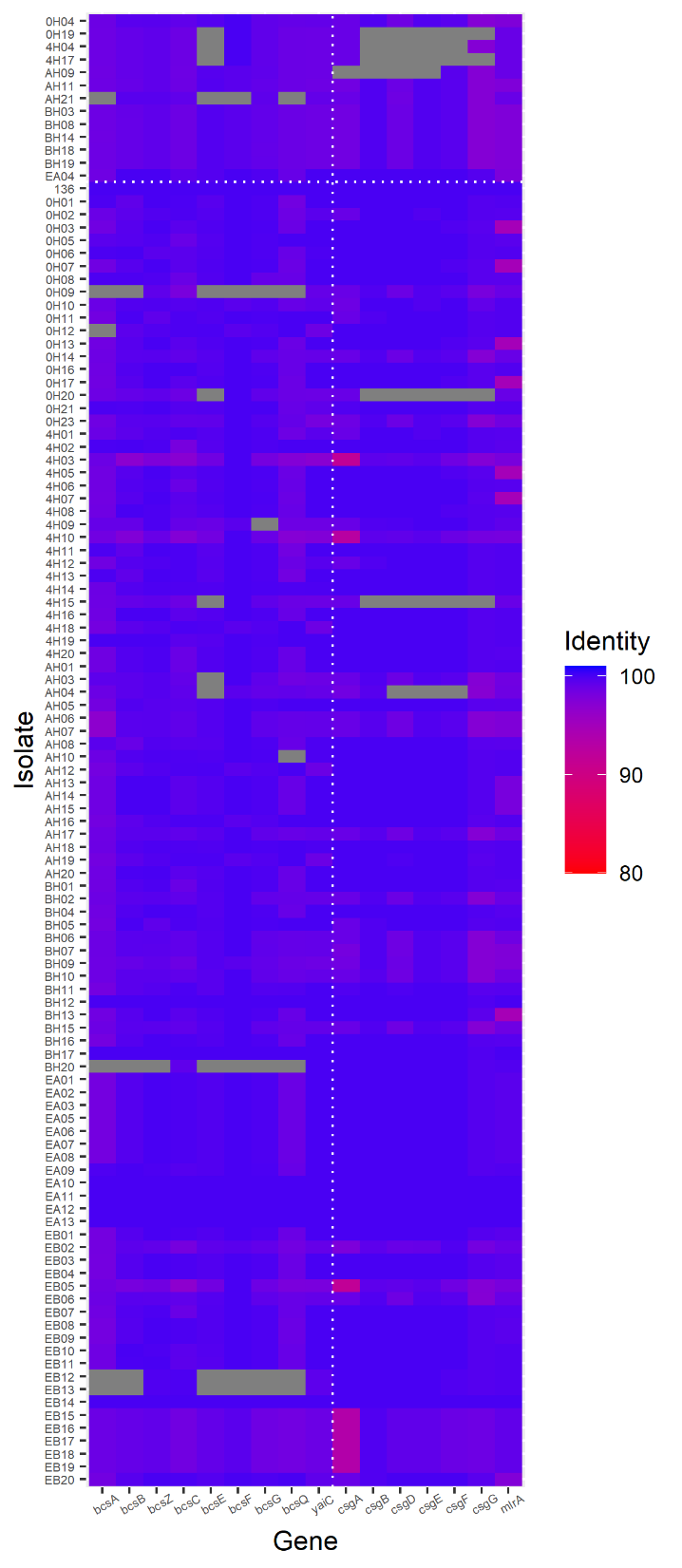

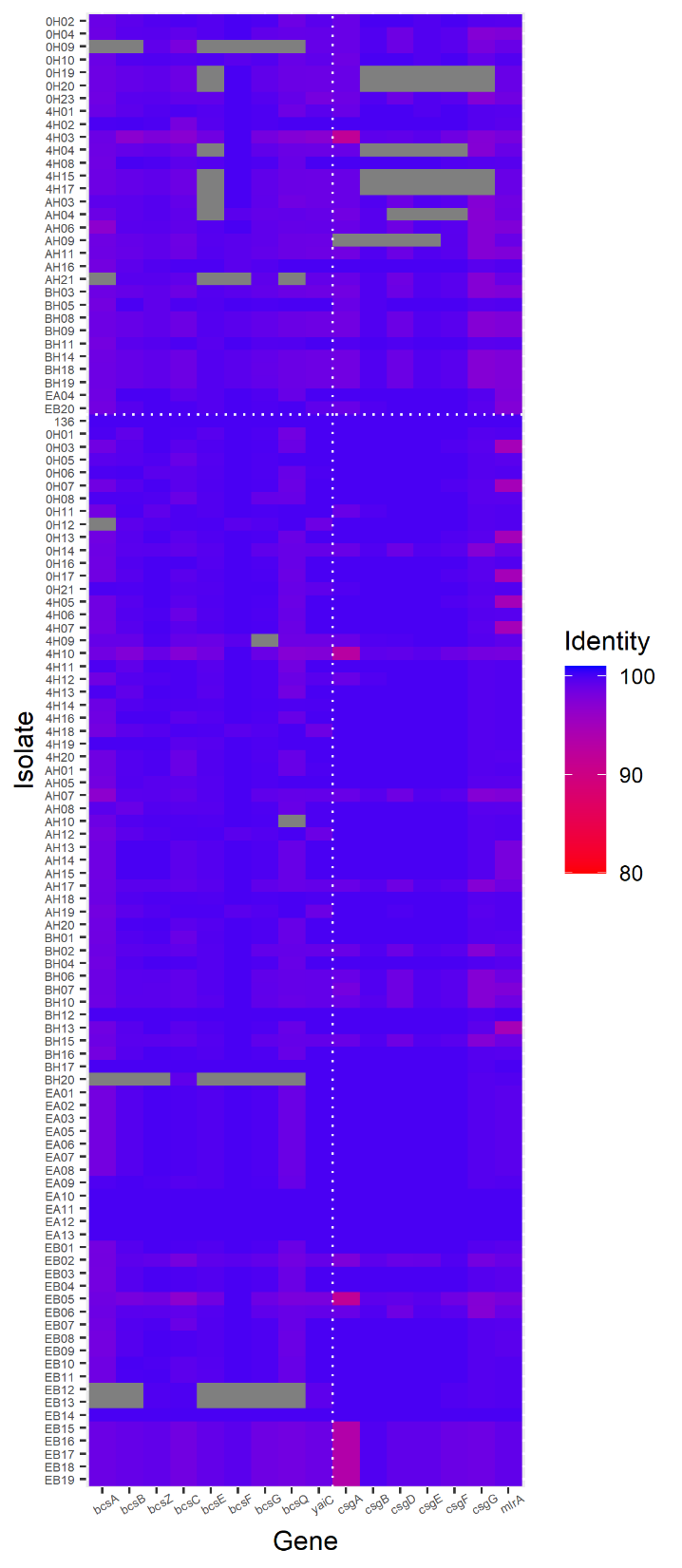


**B**

**A**

**Figure S2.** The presence and extent of similarity of genes involved in cellulose and curli production in *E. coli* genomes (n=114). The data in Figure 6 were replotted for a better visualization of the relationship of cellulose and curli genes and their respective phenotype. The vertical line separates the genes involved in cellulose (left) and curli (right). A horizontal dotted line separates the negative (top) and positive (bottom) phenotypic trait for cellulose (A) and curli (B). The identities between relevant genes in each isolate and in strain 136 are shown in color from red to blue. The grids filled with gray indicate the isolate has 0 or more than one copy of the corresponding gene and hence identity level is not shown.


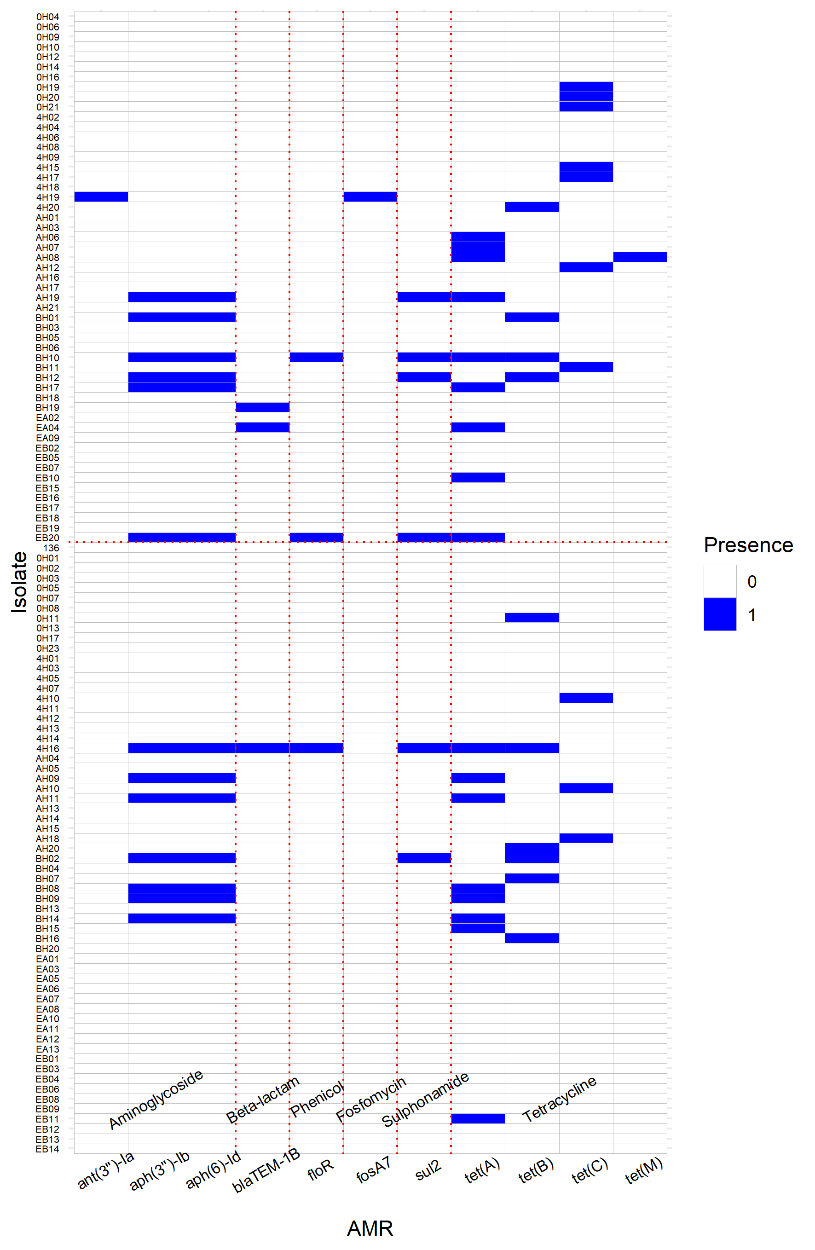


**Figure S3.** Distribution of antimicrobial resistance (AMR) genes in the 114 *E. coli* genomes. The isolates above and below the horizontal red line are non-biofilm formers and biofilm formers, respectively. The grid is color coded for presence (blue) and absence (blank) of a gene.
